# 新辅助免疫治疗及联合化疗在NSCLC中的研究进展

**DOI:** 10.3779/j.issn.1009-3419.2021.102.10

**Published:** 2021-04-20

**Authors:** 思 陈, 泽锐 赵, 浩 龙

**Affiliations:** 510060 广州，中山大学肿瘤防治中心胸外科 Department of Thoracic Surgery, Sun Yat-Sen University Cancer Center, Guangzhou 510060, China

**Keywords:** 肺肿瘤, 免疫治疗, 新辅助, 综述, Lung neoplasms, Immunotherapy, Neoadjuvant, Review

## Abstract

非小细胞肺癌(non-small cell lung cancer, NSCLC)患者在接受标准治疗后仍有较高的复发、转移风险。免疫检查点抑制剂治疗在晚期肺癌患者中获得巨大进展，临床前研究及部分Ⅱ期临床研究为新辅助免疫治疗局部晚期NSCLC提供理论支持和临床证据。本篇综述阐述了免疫治疗的机制及新辅助治疗的理论优势，总结现已发表前期数据和中期分析的多篇临床研究，分析了新辅助免疫单药治疗对比免疫联合化疗的病理缓解率和安全性。NCT02259621、NEOSTAR、LCMC3及ChiCTR-OIC-17013726分别报告了新辅助应用纳武利尤单抗、阿替利珠单抗和信迪利单抗单药的有效性和安全性，同时NEOSTAR双药免疫组的数据显示双药免疫相较于单药可以获得更高的病理缓解率，双药免疫的高不良反应发生率也引起研究者的担忧。NCT02716038、SAKK 16/14和NADIM研究的数据提示新辅助免疫联合化疗的病理缓解率高于新辅助单药免疫抑制剂治疗，且不良反应可耐受。本文进一步阐述了化疗联合免疫抑制剂治疗的作用机制，并就新辅助免疫治疗后的疗效评估进行讨论。

## Abstract (en)

Even patients after standard surgery and adjuvant chemotherapy still have a high risk of recurrence and metastasis. With the success of immunotherapy in advanced non-small cell lung cancer (NSCLC), the application of immunotherapy in locally advanced NSCLC has being investigated to reduce the recurrence and metastasis. Pre-clinical studies and several phase Ⅱ clinical studies had provided theoretical support and clinical evidence for neoadjuvant immunotherapy for NSCLC. This review describes the mechanism of neoadjuvant immuno-chemotherapy, summarizes up-to-date clinical studies, and analyzes efficiency and feasibility of neoadjuvant immune monotherapy or immuno-chemotherapy. Results from four studies (NCT02259621, NEOSTAR, LCMC3 and ChiCTR-OIC-17013726) showed efficiency and feasibility of neoadjuvant anti-programmed cell death 1 (PD-1)/programmed cell death ligand 1 (PD-L1) monotherapy. Neoadjuvant nivolumab plus ipilimumab achieved higher major pathological response rate than nivolumab monotherapy. However, the combination of nivolumab plus ipilimumab led to more severe adverse events as is seen in the NEOSTAR trial. Results from NCT02716038, SAKK 16/14 and NADIM studies suggest that the pathological response rate of neoadjuvant immune-chemotherapy is higher than neoadjuvant immune checkpoint inhibitor monotherapy. This review also elaborates the mechanism of chemotherapy combined with immunotherapy, and discusses the efficacy evaluation after neoadjuvant immunotherapy.

非小细胞肺癌(non-small cell lung cancer, NSCLC)手术后较高的复发率和远处转移风险是制约改善患者远期生存的重要因素。随着免疫治疗在晚期肺癌治疗中取得的巨大成功，新辅助免疫治疗在可切除或潜在可切除的NSCLC患者的初步临床研究所体现出的良好效果更是给学界带来巨大冲击。现有数据显示出新辅助免疫联合化疗应用于NSCLC有良好病理缓解率。本文将对新辅助免疫单药治疗及联合化疗在NSCLC中的运用进行综述。

## 1 新辅助治疗对比辅助治疗的优势

1983年Friedlander在宫颈癌的治疗中首次提出新辅助化疗(neo-adjuvant chemotherapy, NAC)的概念。理论上，新辅助治疗的优势包含：①降低肿瘤负荷使部分不可手术或难以进行根治性手术的患者获得根治性手术可能，相较于化疗、内分泌治疗等全身系统治疗，根治性手术切除是NSCLC现今获得治愈可能的最佳治疗方式，术前新辅助治疗的最主要目的是为更多患者争取根治性手术机会，进一步提高远期生存。因此新辅助治疗对于患者的选择主要集中在Ⅲa期，这部分患者有的肿瘤体积过大或侵及重要器官使手术难度增大，有的伴纵隔淋巴结转移，不推荐一线手术治疗。在进行新辅助治疗后可能使肿瘤体积缩小，降低手术风险，提高手术切除率，或使转移淋巴结转阴，达到降N分期的目的，现也有部分临床研究将患者群体扩展至Ib期-Ⅲa期甚至部分潜在可切除的Ⅲb期患者，以期使更多患者接受根治性手术治疗。②相较于辅助治疗，原发灶存在时可以更直观通过影像学评估疗效。③患者未经历手术创伤，对治疗耐受性好。④更早期开展全身治疗对抗潜在微转移灶，降低转移风险。⑤新辅助治疗后手术标本的病理缓解率可能成为术后生存的预测因子，指导复发高危人群及时进行辅助治疗。

但是在NSCLC中，NAC的应用疗效存在争议。虽然NAC相较于单纯手术可提高生存^[1]^，但是对比辅助化疗，无病生存期(disease free survival, DFS)无明显差异^[2]^。由吴一龙教授牵头的CSLC0501试验中期结果甚至提示接受辅助化疗的患者在3年DFS率上优于接受NAC的患者^[3]^。同样地，近年来新辅助靶向治疗的临床试验结果发现，术前应用靶向治疗对比术前化疗虽然可以提高改善无进展生存期(progression-free survival, PFS)，但在客观缓解率上两者并无明显差异^[4]^。在黑色素瘤及胆囊癌等其他癌种已经发表的临床前和部分小规模临床试验显示，新辅助免疫治疗相比于辅助免疫治疗可能有优势^[5, 6]^。Liu等^[7]^通过三阴乳腺癌的动物模型发现新辅助免疫治疗相较于辅助免疫治疗可显著延长荷瘤小鼠生存期。

新辅助免疫治疗相较于辅助治疗获得更佳疗效的理论假说为：在新辅助免疫治疗时，患者体内大量肿瘤特异性抗原(tumor-specific antigen, TSA)^[8]^，完整的肿瘤引流淋巴结(tumor-draining lymph nodes, TDLN)的存在有利于树突状细胞(dendritic cell, DC)向T细胞呈递TSA^[9]^，因此阻断程序性死亡受体1(programmed cell death 1, PD-1)/程序性死亡受体配体1(programmed cell death ligand 1, PD-L1)通路可使大量活化的肿瘤浸润淋巴细胞(tumor infiltrating lymphocyte, TIL)识别肿瘤细胞，获得更强杀伤肿瘤效果。还有观点^[10]^认为：在接受新辅助免疫治疗后活化的肿瘤特异性T细胞沿循环系统循环时，可识别循环肿瘤细胞表面抗原，消除微转移灶。

## 2 NSCLC的新辅助免疫治疗临床研究

新辅助免疫治疗吸引外科医生及研究者的大量关注，目前免疫检查点抑制剂(immune checkpoint inhibitor, ICI)在NSCLC新辅助治疗的临床试验数量有40余个，且多在招募患者中(表 1)。发布了初步结果或中期分析的试验有10项(表 2)。

**表 1:** 正在进行的NSCLC新辅助免疫相关临床研究 Ongoing trials of neoadjuvant immune-related therapy for NSCLC

	NCT		Regimen	Primary endpoint	Stage	*n*	Estimated completion date	Phase
Neoadjuvant ICI monotherapy	NCT04047186		Nivolumab+S	MPR	Muti-GGO	50	2024/12	2
	NCT03732664		Nivolumab/Pembrolizumab+S	Feasibility and saftey	High-risk resectable NSCLC	40	2027/10	1
	NCT02818920	TOP1501	Pembrolizumab+S+Pembrolizumab	Feasibility and saftey	Ib-Ⅲa	30	2026/3	2
	NCT02938624	MK3475-223	Pembrolizumab+S	Feasibility and saftey	Ⅰ-Ⅱ	28	2021/4	1
	NCT03197467	NEOMUN	Pembrolizumab+S	Feasibility and saftey	Ⅱ-Ⅲa	30	2023/10	2
	NCT02994576	PRINCEPS	Atezolizumab+S	Feasibility and saftey	Ib-Ⅲa	60	2022/12	2
	NCT03030131	IONESCO	Durvalumab+S	Surgical resection	Ib-Ⅲb	81	2019/8	2
	NCT04371796		Sintilimab+S	MPR	Ⅱ-Ⅲa	20	2021/12	2
	NCT04197076		ICI^*^2+S	DFS, pCR	Ⅲa	200	2021/5	
	NCT03853187	DONAN	Durvalumab+S+RT/CT	Feasibility and saftey	Ⅲ	20	2022/4	2
Neoadjuvant ICI combine with chemotherapy	NCT04541251	TOP-LC1210	(Camrelizumab+CT)^*^3	MPR	Ib-Ⅲa	40	2023/9	2
	NCT04144608		(Toripalimab+CT)+S	Surgical resection	Ⅲa or Ⅲb	30	2020/12	2
	NCT04304248	NeoTPD01	(Toripalimab+CT)^*^3	pCR	Ⅲ	30	2026/7	2
	NCT04586465	DYNAPET	(Pembrolizumab+CT)^*^3	MPR、SUV	IIa-Ⅲb	23	2022/6	2
	NCT04379739		Camrelizumab+CT	MPR	Ⅱ-Ⅲa	82	2026/12	2
			Camrelizumab+Apatinib					
Neoadjuvant and adjuvant ICI	NCT04512430		(Atezolizumab+Bevacizumab+CT)+S+ (Atezolizumab q4w^*^6 mon)	MPR	Ⅲa (EGFR+)	26	2026/8	2
	NCT04465968	DEEP_OCEAN	(Durvalumab+RT+CT)+S+ (Durvalumab/RT+CT)	3yr-OS	Ⅲ	84	2030/8	3
	NCT04326153		(Sintilimab+CT)+S+(Sintilimab^*^8+CT^*^2)	2yr-DFS	Ⅲa	40	2022/12	2
	NCT03838159	NADIMII	(Nivolumab +CT)^*^3+S+(Nivolumab^*^1 y)	pCR	Ⅲ	90	2027/9	2
RCT	NCT02998528	CheckMate816	(Nivolumab+CT)+S	EFS, pCR	Ib-Ⅲa	350	2028/11	3
			S+CT					
			(Nivolumab+Ipilimumab)+S					
	NCT03425643	KEYNOTE-671	(Pembrolizumab+CT)^*^4+S+(Pembrolizumab^*^1 y)	EFS, OS	Ⅱ-Ⅲb (T3-4N2)	786	2026/6	3
			NAC+S					
	NCT03456063	IMpower030	(Atezolizumab+CT)+S+(Atezolizumab^*^16)	MPR, EFS	Ⅱ-Ⅲb	450	2024/11	3
			NAC+S					
	NCT03800134	AEGEAN	(Durvalumab+CT)+S	MPR, EFS	Ⅱ-Ⅲ	800	2024/1	3
			NAC+S					
	NCT04025879		(Nivolumab+CT)+S+(Nivolumab)	EFS	IIa (> 4 cm)-Ⅲb (T3N2)	452	2024/9	3
			NAC+S					
	NCT04338620		(Camrelizumab+CT)+S	pCR	Ⅲ (N2)	94	2021/11	2
			NAC+S					
	NCT04379635		(Tislelizumab+CT)+S+(Tislelizumab)	MPR, EFS	Ⅱ-Ⅲa	380	2025/11	3
			NAC+S					
	NCT04422392		(ICI+CT)+S+(ICI+CT)	MPR	Ⅲa (N2)	90	2025/6	2
			NAC+S+CT					
	NCT04061590		Pembrolizumab +S	TIL	Ⅰ-Ⅲa	84	2022/4	2
			(Pembrolizumab+CT)+S					
	NCT04459611	neoSCORE	(Sintilimab+CT)^*^2+S+(CT^*^2+Sintilimab^*^1 y)	MPR	Ib-Ⅲa	60	2023/7	2
			(Sintilimab+CT)^*^3+S+(CT^*^1+Sintilimab^*^1 y)					
	NCT03916627		Cemiplimab+S+(Cemiplimab+CT)	MPR	NSCLC	94	2027/8	2
			(Cemiplimab+CT)+S+(Cemiplimab+CT)					
			NAC+S+(Cemiplimab+CT)					
Neoadjuvant ICI+RT	NCT02904954		Durvalumab+S+Durvalumab^*^1 y	MPR	Ib-Ⅲa	60	2022/4	2
			(Durvalumab^*^3+RT)+S+(Durvalumab^*^1 y)					
	NCT03217071	PembroX	Pembrolizumab+S	TIL	Ⅰ-Ⅲa	40	2021/12	2
			(Pembrolizumab+RT)+S					
	NCT03237377		(Durvalumab+RT)+S	Feasibility and saftey	Ⅲa	32	2021/9	2
			(Durvalumab+Tremelimumab+RT)+S					
	NCT04245514	SAKK 16/18	(Durvalumab^*^1+CT^*^3+RT)+S+ (Durvalumab^*^13 *q4w*)	EFS	T1-4 (> 7 cm) N2	90	2025/3	2
Data updated to November 1, 2020. RT: radiation therapy; ICI: immune checkpoint inhibitor; S: surgery; CT: chemotherapy; NAC: neoadjuvant chemotherapy; MPR: major pathologic response; DFS: diease-free survival; pCR: pathologic complete response; EFS: event-free survival; TIL: tumor infiltrating lymphocyte; OS: overall survival; NSCLC: non-small cell lung cancer; SUV: standard uptake value; RCT: randomized controlled trial; RFS: relapse-free survival; TMB: tumor mutation burden; NA: not available; EGFR: epidermal growth factor receptor.								

**表 2:** NSCLC新辅助免疫治疗临床试验 Trials of neoadjuvant immunotherapy for NSCLC

		*n*	Stage	Surgical resection	Regimen	MPR	pCR	ORR	Potential predictor	Pathological downstage	> 3 TRAEs	Survival
ICI+S	NCT02259621^[11]^	22	Ⅰ-Ⅲa	21 R0:20	Nivolumab^*^2+S	9(45%)	2 (10%)	2 (10%)	TMB	8 (40%)	1 (5%)	18 mon_RFS: 73%
	ChiCTR-OIC-17013726^[41]^	49	Ia–Ⅲb	37	Sintilimab+S	15 (40%)	6 (16%)	8 (20%)	PET-CT SUV downregulate > 30%	14 (29%)	4 (10%)	NA
	LCMC3 (NCT02927301) ^[13]^	101 /180	Ib-Ⅲa	90	Atezolizumab^*^2 +S	15 (18%)	4 (5%)	NA	NA	NA	4 (4%)	NA
	IONESCO	46	Ib-Ⅲa	44 R0:41	Durvalumab^*^3+S	8 (17%)	3 (7%)	4 (9%)				1yr_RFS: 78.2%; 1yr_OS: 89.1%
	PRINCEPS (NCT02994576)	30	Ⅰ-Ⅲa	30 R0:29	Atezolizumab^*^1 +S	4 (13%)	0	2 (7%)	PD-L1	NA		NA
	NEOSTAR (NCT03158129) ^[15]^	23	Ⅰ-Ⅲa	21	Nivolumab+S	4 (17%)	2 (9%)	NA	NA	NA	1 death	NA
(nivol+ipi) +S		21		16	(Nivolumab+ Ipilimumab)+S	6 (29%)	4 21%)					
(ICI+CT) +S	NCT01820754 (TOP1201) ^[20]^	24	Ib-Ⅲa	13	CT^*^1+(Ipilimumab +CT)^*^2+S	NA	NA	14 (58%)	NA	NA	11 (46%)	mOS: 29.2 mon
	NCT02716038^[18]^	30	Ib-Ⅲa	29 R0:26	(Atezolizumab +CT)^*^2+S	17 (57%)	10 (33%)	19 (63%)	NA	19 (63%)	15 (50%)	mDFS: 17.9 mon
	SAKK 16/14 (NCT02572843)^[19]^	68	Ⅲa (N2)	55	(CT^*^3+Durvalumab^*^2) +S	33 (60%)	10(18%)	NA	NA	37 (67%)	59 (88%)	1yr_EFS: 73.3%
	NADIM (NCT03081689) ^[34]^	46	Ⅲa (N2)	41	(Nivolumab+CT)+S	34 (83%)	26 (63%)	35 (76%)	PD-L1	29 (63%)	16 (34%)	2yr_PFS: 77.1%; 2yr_OS: 89.9%
PET：positron emission tomography; TRAE：Treatment-related adverse events												

### 2.1 新辅助免疫单药治疗

在新辅助免疫治疗探索初期，开展了多项免疫单药新辅助免疫治疗的临床试验。2018年，Forde等^[11]^报道了21例应用2程纳武利尤单抗单药新辅助免疫治疗的Ⅰ期-Ⅲa期NSCLC患者(NCT02259621)，其中20例患者达到R0切除，主要病理缓解(major pathologic response, MPR)率为45%，2例患者(10%)达到病理完全缓解(pathologic complete response, pCR)。这一结果的发布给予研究者们极大的鼓舞。在其他临床试验中，免疫单药新辅助治疗的MPR率则有所下降。NEOSTAR的纳武利尤单药组的MPR率为17%，pCR为9%^[12]^。2020年度世界肺癌大会更新的LCMC3的中期数据显示在已公布的符合入组条件且接受手术治疗的159例患者数据中，阿替利珠单抗新辅助治疗后MPR率为21%，pCR率为7%^[13]^。中国医学科学院肿瘤医院牵头的信迪利单抗单药新辅助治疗Ia期-Ⅲb期NSCLC患者的Ⅱ期研究(ChiCTR-OIC-17013726)初步数据显示在37例接受根治性手术的患者中，15例患者(40.5%)达到MPR，其中6例(16.2%)患者达到pCR^[14]^。新辅助免疫单药治疗的MPR率在17%-45%。安全性方面显示未明显延误手术时机，相关不良反应在可耐受范围。Forde等^[11]^的研究中没有患者因新辅助治疗延误手术时机，LCMC3研究159例手术患者中仅有4例因治疗相关不良反应延误手术，最终也完成手术治疗，术前术后3级以上不良反应发生率分别为6%和13%。信迪利单药研究不良反应发生率为10%，主要表现为肺炎、肺部感染等。

### 2.2

新辅助PD-1/PD-L1抑制剂联合细胞毒性T淋巴细胞相关蛋白4(cytotoxic T lymphocyte-associated antigen-4, CTLA-4)抑制剂NEOSTAR(NCT03158129)是直接对比PD-1抑制剂纳武利尤单抗或PD-1抑制剂联合CTLA-4抑制剂伊匹木单抗新辅助治疗Ⅰ期-Ⅲa期(单站N2)期NSCLC的疗效和安全性的Ⅱ期临床研究^[12, 15]^。在入组的44例患者中有23例接受PD-1单药治疗，另外21例接受双免疫联合治疗。34例患者接受了手术切除，MPR率单药组为17%(4/21)，双免组为29%(6/16)，pCR率单药组为9%(2/21)，双免组为21%(4/16)。单药组常见的不良反应有疲劳、皮疹、贫血等，常见的3级-5级不良反应有高镁血症、低氧、肺炎、局限性肺炎。双免组的不良反应有皮疹、疲劳、恶心等，常见的3级-5级不良反应有腹泻、低钠血症。单药组术后并发症有胸腔漏气、支气管胸膜瘘、肺炎等，其中1例患者因支气管胸膜瘘去世，双药组的术后并发症主要为胸腔漏气。在该研究中单药组与双药组的不良反应无明显差异，但在黑色素瘤研究中，双免方案的3级-4级不良反应率为55%，远高于单药方案的16.3%^[16]^。因此，双免新辅助方案治疗NSCLC带来的毒性反应仍需要进一步评估^[17]^。

### 2.3 新辅助免疫联合化疗

目前开展的NSCLC新辅助免疫治疗相关临床试验多为免疫联合化疗方案。这些已报道的研究结果显示了其相较于免疫单药新辅助治疗更高的MPR率。

一项新辅助阿替利珠联合化疗治疗Ib期-Ⅲa期NSCLC的临床研究(NCT02716038)获得了不错的病理缓解率^[18]^，MPR和pCR率分别为57%(17/30)和33%(10/30)。SAKK 16/14研究(NCT02572843)研究了3周期顺铂/多西他赛NAC后序贯2周期度伐利尤单抗治疗Ⅲa(N2)期NSCLC患者的应用价值，其初步研究结果显示序贯新辅助免疫可在NAC后进一步提高影像学缓解率(从44.8%提升至58.1%)^[19]^。而在55例接受手术切除的患者中，MPR率为60%(33/55)，pCR率为18.2%(10/55)。37例(67.3%)患者观察到术后淋巴结分期下降。1年无事件生存(event-free survival, EFS)率为73.3%。NADIM研究为一项探索术前纳武利尤单抗联合紫杉醇、卡铂化疗在表皮生长因子受体(epidermal growth factor receptor, EGFR)及间变性淋巴瘤激酶(anaplastic lymphoma kinase, ALK)阴性、Ⅲa期(N2/T4N0)潜在可手术切除NSCLC患者应用价值的Ⅱ期临床试验，其报道MPR率高达83%(34/41)，且有63%(26/41)患者达到pCR，2年PFS率为77.1%，2年总生存期(overall survival, OS)率为89.9%。

虽然免疫联合化疗相较于免疫单药治疗的不良反应发生率上升，但也在可耐受范围内。SAKK 16/14中发生3级以上不良反应的患者比例为88.1%，较高的不良反应发生率是否与其试验设计3程化疗后序贯2程免疫治疗存在关系仍需验证。其他两项同期免疫联合化疗的临床试验3级以上不良反应率分别为50%^[18]^和46%^[20]^，没有出现治疗相关死亡。

## 3 新辅助免疫治疗联合化疗的基础研究

对比新辅助免疫单药或免疫双药治疗，免疫联合化疗似乎可达到更高的MPR率。化疗联合免疫治疗产生更好疗效的原因可能是化疗在直接杀伤肿瘤细胞的同时，也可参与机体免疫反应的调节，主要体现在：(1)调节免疫细胞数量：①杀伤肿瘤细胞释放大量TSA。化疗一方面可刺激肿瘤细胞表达TSA，例如癌胚抗原(carcinoembryonic antigen, CEA)^[21]^和肿瘤睾丸抗原(cancer-testis antigens, CTA)^[22]^，另一方面对于肿瘤细胞的直接杀伤作用在肿瘤细胞死亡的同时也可释放大量TSA。②化疗药物可使肿瘤细胞表面的钙网蛋白表达增加，上调肿瘤细胞表面Ⅰ类主要组织相容性复合体(major histocompatibility complex-I, MHC-I)，促进TSA向CD8^+^细胞毒性T细胞的呈递^[23]^，促进T细胞活化。临床前研究显示，部分化疗药物可以通过影响肿瘤微环境中的免疫细胞发挥抗肿瘤作用，例如培美曲塞可以增加TIL的含量^[24]^，提高PD-1/PD-L1抑制剂的疗效。③部分化疗药物作用于肿瘤细胞使其分泌免疫相关因子，这些因子通过激活DC细胞分化成熟、Ⅰ型干扰素应答和T细胞募集等途径促进免疫作用^[25]^。④减少抑制性免疫细胞数量：吉西他滨和顺铂可特异性减少化疗敏感免疫抑制细胞，如髓系抑制细胞(myeloid-derived suppressor cells, MDSC)、调节性T细胞(regulatory T cells, Treg)等细胞^[26, 27]^，在应用免疫联合多西他赛+顺铂的NSCLC患者的外周血观察到Treg细胞比例的下降^[28]^。在机体内，免疫联合化疗通过上述多种机制一同发挥协同作用，增强抗肿瘤效果。在小鼠实验中，吉西他滨一方面可以促进TSA释放，增强CD8^+^ T细胞活性，另一方面也可以显著降低MDSC含量。(2)抑制肿瘤的免疫逃逸机制：①化疗可以上调肿瘤细胞表面的共刺激因子受体(B7-1)，下调抑制因子受体(PD-L1)^[23]^。②免疫治疗后激活的免疫细胞和细胞因子同样可以增强肿瘤细胞对化疗的敏感性，使肿瘤细胞对于T细胞通过Fas/Fas-1及Gramzyne B途径介导的杀伤作用更为敏感。

部分临床前研究^[29]^显示，在免疫治疗前注射化疗药物环磷酰胺或紫杉醇可以削减Treg数量，增强免疫相关T细胞应答，提高小鼠的无肿瘤生存期。一项小鼠研究提示，每日注射低剂量环磷酰胺相比一次性大剂量治疗T细胞应答更强，这可能与大剂量化疗对淋巴细胞的无差别杀伤有关^[30]^。这提示我们在免疫治疗联合化疗时，化疗药物的剂量及疗程设置是否需要作出相应调整。

## 4 新辅助免疫治疗的疗效评估

在抗肿瘤治疗前后通过影像学的对比显示治疗疗效，根据实体瘤疗效评价标准(Response Evaluation Criteria in Solid Tumors, RECIST)标准，客观缓解率(objective response rate, ORR)可作为评估患者预后的指标。阿替利珠联合化疗的一项研究^[18]^显示MPR与RECIST标准的ORR存在显著相关(*P*=0.002, 2)。但是，很多研究提示ORR并不能完全预测患者OS。Bichard^[31]^认为肿瘤应答率与预后无关。在部分NAC的研究中观察到了明显的肿瘤退缩但术后生存期对比单纯手术并未获得显著延长，如EORTC08012研究诱导化疗后ORR率为49%，但是术后5年OS和2年PFS对照单纯手术组均无明显差异^[32]^。针对免疫治疗产生的特殊影像学表现——假进展，Humbert等^[31]^认为在治疗后3个月的正电子发射计算机断层显像(positron emission tomography-computed tomography, PET-CT)影像学数据较治疗后7周更能反映患者真实情况。在高树庚教授团队报道的研究中，新辅助免疫治疗前后PET检查中标准摄取值(standard uptake value, SUV)值下降比例似乎是可以预测疗效的潜在指标^[14]^。为了鉴别免疫治疗后的假性进展与疾病进展(progressive disease, PD)的情况，iRECIST标准亦被提出^[33]^。有趣的是，在已公布的两项新辅助免疫联合化疗的临床试验中，均未观察到假性进展的事件^[18, 34]^。

OS是被广泛接受的评估抗肿瘤获益的“金标准”。但是要得到可信的OS需要花费很长时间观察到终点事件的发生，既往关于辅助及新辅助的临床研究需要9年-13年的时间来报道最终生存结果^[35]^。新辅助免疫后手术切除组织发现出现pCR是新辅助治疗的理想效果。大量研究提示pCR与OS存在相关性^[36]^。一项研究^[37]^提示NAC后获得pCR的患者其远期生存与Ib期NSCLC相仿。如前所述，目前进行的大部分新辅助免疫治疗研究的研究终点包括MPR，将其作为患者从新辅助治疗中取得长期生存获益的替代指标。NADIM研究发现病理评估达到MPR以上患者2年OS率显著优于未达到MPR的患者(*P*=0.002)，但不含pCR的MPR患者的OS率与未达到MPR的患者无明显差异^[34]^。因此对于MPR的设定需要更多的临床数据支持。有研究者建议在对患者进行治疗后病理评估时采用剩余肿瘤占瘤床的百分比的定量数据，而不是简单划分为MPR或非MPR的分类数据^[35]^。也有近期研究^[38]^提示，NAC后达到MPR的患者中，可能仅有原发灶与淋巴结病灶均完全降期(MPRypN0)的部分亚组能获得生存获益。阿替利珠单抗联合化疗中达到MPR的患者中位DFS为34.5个月，未达到MPR患者的中位DFS为14.3个月(*P*=0.71)，目前随访时间尚短，不能获得统计学意义差异。MPR与OS的关系仍需大量临床数据的验证，以及对于MPR的界定需要更细致的探究与规范。

## 5 新辅助免疫治疗后的手术安全性问题

新辅助免疫治疗后的手术安全性也是外科医生关注的焦点，但是对此缺乏客观性评价指标。目前一般从是否手术延期、手术时长、术中失血量、手术方式(胸腔镜手术或开放手术或中转开胸)、手术术式(肺叶切除术、楔形切除术、全肺切除术)及住院时间等方面进行评估。

现有的数据显示新辅助免疫单药及联合化疗并未导致大量手术延期事件的发生。NADIM研究中5例入组患者未完成手术，其中2例患者拒绝手术，3例患者因不符合手术适应征或存在手术禁忌证而放弃手术治疗。NEOSTAR研究的结果显示，自末次治疗到手术的中位时间为31天(21 d-87 d)，单药组3例(14%)患者和双免组5例(31%)患者等待手术时间大于42 d^[15]^。其他几项临床研究及Bott等^[39]^于2019年报道的20例接受新辅助纳武单抗后行肺部手术切除患者的围术期安全性数据，结果均提示新辅助免疫治疗并不推迟接受手术的时机。

对于手术难度产生影响的主要原因有：①肿瘤体积大或侵及重要组织器官; ②组织纤维化，血管脆性增加，淋巴结清扫难度增加^[40]^。TOP1201的结果显示不论是在接受NAC或NAC联合免疫治疗的患者中最常见的并发症为动脉纤维化，NAC的患者中出现动脉纤维化的比例为14%，而新辅助联合治疗中的发生率为8%。两种治疗方法间对比没有明显手术并发症的差异。与之相似的，Stiles等在2019年度美国胸外科年会(American Association for Thoracic Surgery, AATS)上报道了24对接受新辅助免疫对比NAC的对比研究，结果提示两组在围手术期心肺并发症发生概率上无明显差异。LCMC3的研究^[13]^同样显示在90例患者中，有20例(22.2%)患者出现广泛肺门纤维化。此外，接受新辅助免疫治疗后达到MPR的患者更容易出现肺门结构纤维化改变。

Bott的研究在13例拟行微创胸腔镜手术的病例，有54%的病例需要中转开胸完成肿瘤切除，提示了新辅助免疫治疗对于手术难度的影响。NEOSTAR的数据显示27例行开胸手术，7例行胸腔镜手术，3例经机器人手术治疗。未显示中转开胸率。中位手术时间为147 min(71 min-315 min)，中位失血率为100 mL(50 mL-1, 000 mL)，中位住院时间4 d(1 d-18 d)^[15]^。可见虽然部分手术难度增加，但术后并发症未见明显增加。而在LCMC3的更新数据中，54%的患者可接受微创手术，中转开胸率为15%，且全部入组人群中仅有3%出现术中支气管/血管并发症。可见对于新辅助治疗产生组织纤维化的机制和其对手术的影响需要更为量化的标准进行评估和研究。

## 6 NSCLC新辅助免疫治疗标准化面临的挑战

现有的临床数据展示了新辅助免疫联合化疗在局部晚期NSCLC患者中的巨大潜力，免疫联合化疗现已在NSCLC中广泛应用，但对于免疫治疗联合化疗是协同作用或单纯叠加作用，相关机制尚不清楚，需要基础研究进一步探究。免疫联合化疗的疗程设计也引起部分研究者的注意，同时行联合治疗、同一疗程化疗后行免疫治疗或是数程化疗后序贯免疫治疗？剂量应当减量或维持常规剂量？新辅助治疗后与手术间隔多久手术并发症最低？这些问题都有待解答。对于部分应用免疫治疗出现严重不良反应的患者，是否存在生物学指标可以在治疗前提示不良反应的发生，以降低不良反应发生率。同时是否存在相应疗效标注物可以在治疗前筛选出高获益人群，使患者利益最大化。后续临床研究的数据需明确病理评估指标MPR与pCR和生存指标DFS及OS的关系，此外，新辅助免疫治疗后经手术切除患者的最佳辅助治疗模式仍有待探索。
